# Seed Characteristics and Nutritional Composition of Pine Nut from Five Populations of *P. cembroides* from the States of Hidalgo and Chihuahua, Mexico

**DOI:** 10.3390/molecules24112057

**Published:** 2019-05-30

**Authors:** José Valero-Galván, Margarita Reyna-González, Perla Anneth Chico-Romero, Nina del Rocío Martínez-Ruiz, José Alberto Núñez-Gastélum, Abigail Monroy-Sosa, Eliel Ruiz-May, Raquel González Fernández

**Affiliations:** 1 Instituto de Ciencias Biomédicas, Departamento de Química-Biológicas, Universidad Autónoma de Ciudad Juárez, Chihuahua, Chihuahua C.P. 32310, México; m_reyna_g@outlook.com (M.R.-G.); al128912@alumnos.uacj.mx (P.A.C.-R.); nmartine@uacj.mx (N.d.R.M.-R.); jose.nunez@uacj.mx (J.A.N.-G.); raquel.gonzalez@uacj.mx (R.G.F.); 2 Secretaría de Medio Ambiente y Recursos Naturales, Av. Progreso N° 3, Planta Alta, Col. del Carmen, Del. Coyoacán, Ciudad de México C.P. 04100, México; abi_monroy@yahoo.com.mx; 3 Red de Estudios Moleculares Avanzados, Clúster Científico y Tecnológico BioMimic^®^, Instituto de Ecología A.C. (INECOL), Carretera Antigua a Coatepec No. 351, Congregación el Haya, Xalapa, Veracruz C.P. 91070, México; eliel.ruiz@inecol.mx

**Keywords:** pine nut, seed variability, seed proximal composition, seed population variability

## Abstract

The aim of this study was to analyze the seed characteristics and nutritional composition of five pine nut *P. cembroides* samples from two Mexican states. Morphometry, proximal composition, phenolic compounds, and antioxidant capacity were determined. Samples differed in several morphometric trails, but important differences were documented between SMCH and JCZH samples from Hidalgo State. JCZH and FMH had the highest contents of water, lipids, protein, flavonoids, and antioxidant activity, while CMCC population from Chihuahua State had presented the highest content of ash and carbohydrates. Morphometry and chemical composition data were subjected to clustering analysis. This analysis showed that SMCH and LFCH from Hidalgo State were well separated from the JCZH and FMH populations from Hidalgo State, which showed a strong similarity between them, while the CMCC from Chihuahua State was the most distant population. Principal components analysis showed that the variables that strongly contributed to PC1 were the antioxidant activity determined by FRAP assay, flavonoids, and water content. These data have provided biochemical markers that could help to establish phylogenetic associations between populations, and also to reveal potentially account as an alternative source for dietary nutrition.

## 1. Introduction

In Mexico, fifteen species of *Pinus* sp. produce edible nuts. These developed plant communities of three represent an essential resource for the populations and the surrounding environment of these zones because they play roles in maintaining the hydrological regime of the basins, modulating local microclimates, and providing shelter and food for associated wildlife and domestic livestock. *Pinus* species are also sources of wood, resins, and seeds for a wide range of applications.

In Mexico, the forest with the greatest distribution and territorial extension is formed mainly by *Pinus cembroides* Zucc. [1]. The distribution of this specie included the states of Aguascalientes, Baja California, Chihuahua, Coahuila, Durango, Guanajuato, Hidalgo, Jalisco, State of Mexico, Nuevo Leon, Puebla, Querétaro, Tamaulipas, San Luis Potosí, Sonora, Tamaulipas, Tlaxcala, Veracruz, and Zacatecas [2,3,4]. In the Mexican highlands, *P. cembroides* occupies transition zones due to its ability for adaptation and remarkable resistance to adverse climatic and poor soil conditions, in such zones it can grow in shallow soils with pH values ranging from 4 to 8. These characteristics make *P. cembroides* trees suitable for reforestation of semiarid or eroded arid, habitats which have been extensively impoverished historically by intensive agriculture and cattle feed activities [5]. Furthermore, the populations of *P. cembroides* are being threatened by global warming, fire, pests, diseases, and anthropogenic activities, altogether contributing to massive deterioration of the inhabiting ecosystems [6,7]. In the states of Hidalgo and Chihuahua, one of the most important species in the production of pine nut is *P. cembroides* [6]. The population of *P. cembroides* located in these areas has been subject to self-consumption and trading to neighboring communities. However, as it also happens in other regions of Mexico, the production of pine nuts has many limitations: (i) it is carried out in the rustic format, (ii) the collectors do not have an adequate training, (iii) a lack of technical management of the stands, and (iv) lack of a proper system of quality control of the collected seeds [6].

Valuable nutrients and functional phytochemicals of edible pine nut of *P. pinea* (i.e., 5.1% moisture, 31.6% protein, 44.9% fat, 13.9% carbohydrate and 4.5% ash) [8], *P. halepensis* (i.e., 7.8% moisture, 26.6% protein, and 36.7% fat) [9], *P. pinaster* (i.e., 8.2% moisture, 16.2% protein, and 24.1% fat) [9], *P. canariensis* (i.e., 8.6% moisture, 16.7% protein, and 23.9% fat) [9], and *P. maximartinezii* (i.e., 5% moisture, 31% protein, and 42% fat) [10] have already been documented, but studies of physicochemical and nutritional/phytochemical characterization of Mexican pine nuts are very scarce. The composition of pine nuts shows variation among species and even some subspecies depending on two principal factors, i.e., geographical range and climatic conditions [11,12,13]. It is noteworthy that the consumption of the pine nuts has several effects on consumer health such as the reduction of cardiovascular risk factors by their cardioprotective and antioxidant capacity [14,15,16,17,18].

Lastly, pine nut characterization is important due to health, commercial, and sanitary concerns. However, only a few studies have been carried out analyzing the morphology of *P. cembroides*. Morphological variations of pine nut of *P. cembroides* were referred to the number of seeds per cone or to the percentage of developed and aborted seeds and the percentage of germination were studied in relation to the year of harvest [19]. Likewise, the seed coat composition of pine nut of several phenotypes of *P. cembroides* from the central region of Veracruz has shown statistically significant differences in the composition of fatty acids such as the myristic (3.4–9.1%), oleic (36.7–47.2%), and linoleic (32.9–44.5%) acids [20]. Besides, the dehulled seeds of *P. cembroides* in the United States were a good source of oil (64%), containing high levels of oleic (47%) and linoleic (41%) acids, and only 10% saturated acids [21]. However, the proximal composition, phenolic content and antioxidant activity of pine nut of *P. cembroides* remain unknown. In order to implement a program of genetic improvement through the selection and reproduction of trees and provenances in the states of Hidalgo and Chihuahua, Mexico, it is necessary to characterize the desirable characteristics of the pine nut, such as (i) cones with seeds with greater size, (ii) seeds with thin testa, and (iii) seeds with higher nutritional content. The objective of this study was to make a description of morphometry, the proximal composition, the content of phenolic compounds, and the antioxidant capacity of pine nut from five populations of *P. cembroides*, distributed in the two states above-mentioned.

## 2. Results

### 2.1. Pine Nut Morphometric Analyses

Until now, few studies of *P. cembroides* variability based in the morphological and chemical composition of pine nut have been conducted in the forests of the states of Hidalgo and Chihuahua, Mexico. In the present study, we assessed the natural variability of *P. cembroides* by making a comparison of morphological traits and chemical composition of the seed from five different populations located in the two mentioned states. The results showed statistically significant differences among populations (Table 1). San Miguel Tlazintla, Cardonal, Hidalgo (SMCH) and Jagüey Colorado, Zimapán, Hidalgo (JCZH) populations presented the highest values in unshelled seed (i.e., weight, length, and width), seed coat weight, and shelled seed (i.e., megagametophyte weight, length, width, area, and perimeter) (Table 1); while the La Florida, Cardonal, Hidalgo (LFCH) and Fontezuelas, Metztitlán, Hidalgo (FMH) populations presented intermediate values of seed trail, and finally, the Curvas de Malpaso, Cuauhtémoc, Chihuahua (CMCC) population had the lowest values.

**Figure 1:**
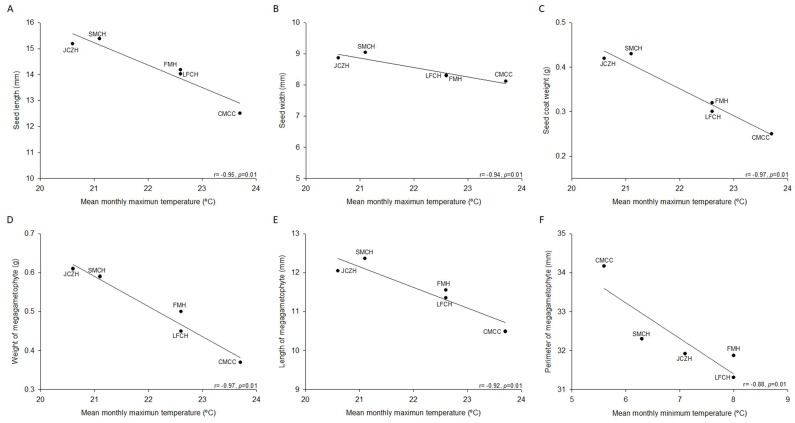
Correlation between morphological characteristics and climatic data. (**A**) Seed length. (**B**) Seed width. (**C**) Seed coat. (**D**) Weight of megagametophyte. (**E**) Length of megagametophyte correlated whit the mean monthly maximum temperature. (**F**) Perimeter of megagametophyte correlated with the mean monthly minimum temperature. Pearson’s correlation coefficient is indicated with a level of significance (*p* ≤ 0.05). Population names are indicated in Table 4.

The difference found in the values in morphometry of seeds among the five populations was statistically significant; this could be associated with the difference in the ecological environment as well as the genotypic and phenotypic variability of this species of pine. In this direction, we correlated the geographical and climatic data of the location of population included in our study. The results showed that seed length (r= −0.86, *p* =0.05) and megagametophyte weight (r = −0.86, *p* = 0.05) were inversely correlated with latitude range of seed source, but positively correlated with the megagametophyte perimeter (r = 0.95, *p* = 0.01). Furthermore, longitude range of seed source showed a direct correlation with seed length (r = 0.87, *p* = 0.05) and megagametophyte weight (r = 0.87, *p* = 0.05) and an inverse correlation with the megagametophyte perimeter (r = −0.95, *p* = 0.01). Additionally, seed length, seed width, seed coat weight, megagametophyte weight, and length showed a statistically significant inverse correlation with mean monthly maximum temperature (Figure 1A–E), while the mean monthly minimum temperature was inversely correlated with a perimeter of megagametophyte (Figure 1F). Thereby, populations located geographically in the northern areas would be expected to have higher pine nut morphometry and weight than southern populations. Nonetheless, these parameters would decrease accordingly with the overall temperature of each provenance.

### 2.2. Chemical Composition of Pine Nut

**Figure 2:**
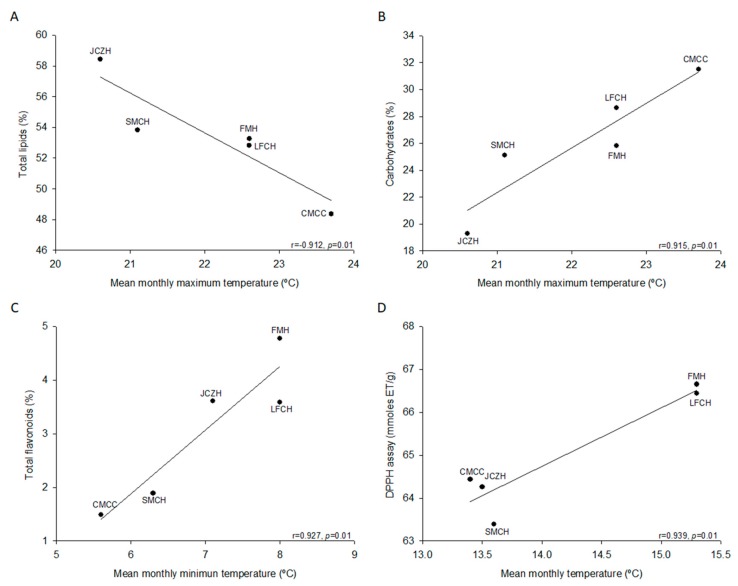
Correlation between chemical composition and climatic data. (**A**) Total lipids. (**B**) Carbohydrates correlated with mean monthly maximum temperature. (**C**) Total flavonoids correlated with the mean monthly minimum temperature. (**D**) DPPH assay correlated with the mean monthly temperature. Pearson’s correlation coefficient is indicated with a level of significance (*p* ≤ 0.05). Population names are indicated in Table 4.

Statistically significant differences were observed for the contents of moisture (*p* = 0.001), ash (*p* = 0.001), total lipids (*p* = 0.05), protein (*p* = 0.001), carbohydrates (*p* = 0.05), and fiber (*p* = 0.001) (Table 2). Seeds from FMH population had the highest humidity, while the population from CMCC had the lowest values. Ash content was higher for the population of CMCC, while LFCH presented the lowest ash content. Total lipid content ranged from 58.4% (JCZH) to 48.4% (CMCC). The protein content of pine nut varied from 15.7% (LFCH) to 19.1% (JCZH). On the other hand, carbohydrates from CMCC presented the highest values, while JCZH presented the lowest ones. The difference in the values found, in chemical composition among the five population was statistically significant, and this could also be related to the difference in the ecological condition, as well as genotypic and phenotypic variability. In this direction, data were also correlated with the geographical and climatic data of population location. The results showed no statistically significant differences with the geographical location. However, when location was correlated with the climatic data, the mean monthly maximum temperature was an inverse correlation with total lipids (Figure 2A) and was positively correlated with carbohydrates (Figure 2B).

### 2.3. Total Phenolics and Flavonoids

Currently, the content of phenolic and flavonoid compounds found in fruits and plant seed, particularly in nuts, has emerged as desirable characteristic for consumption as part of the human diet given their diverse benefits of these bioactive molecules have been demonstrated to act as antioxidative, anticarcinogenic, antihypertensive, anti-inflammatory, antiallergic, and antifungal activities. The analysis of total phenolic contents demonstrated that seed of *P. cembroides* of the states of Hidalgo and Chihuahua could be a good source of phenolic compounds. These results demonstrated a variation from one population to another from 7.8 (SMCH) to 4 (LFCH) mg GAE 100 g^−1^ (Table 2). The analysis for total flavonoid content demonstrated a variation from 4.8 (JCZH) to 1.5 (CMCC) mg CE 100 g^−1^ (Table 2). We could not observe statistically significant correlation when compared the phenolic and flavonoid compounds of seeds to geographical location. However, when these were correlated with the climatic data, the mean monthly minimum temperature was positively correlated with flavonoids (Figure 2C).

### 2.4. Antioxidant Activity

In general, several methods have been used to assess the total antioxidant capacity of pine nuts. In this study, we determined the antioxidant activity by three different methods. The DPPH quenching value confirmed that seeds of *P. cembroides* presented a quenching capacity and a hydrogen donor capacity, and it slightly oscillated among the population from 63.4 to 66.7 mmol TE g^−1^. The FRAP assay measures the ability of the sample to reduce Fe^3+^ (ferric ion) to Fe^2+^ (ferrous ion) in the presence of antioxidants. This methodology also showed slight variation among the population from 21.9 to 26.2 mmol TE g^−1^. Finally, the ABTS^++^ assay measures the capacity of the radical to donate a proton, which allows the analysis of hydrophilic and lipophilic compounds. Using this methodology, results showed a wide variation from 10.9 to 28.2 mmol TE g^−1^ among populations. However, as consensus the three assays for measuring the antioxidant activity showed that FMH presented the highest activity, while the population of SMCH presented the lowest antioxidant capacity as determined by DPPH and ABTS methodologies, and CMCC by FRAP assay (Table 2). We could not observe a significant correlation between parameters associated with the antioxidant activity of seed and the geographical location. However, when the antioxidant activity of the seeds was correlated with the climatic data, the mean monthly temperature was positively correlated with the antioxidant activity determined by DPPH assay (Figure 2D).

### 2.5. Cluster Analysis

**Figure 3:**
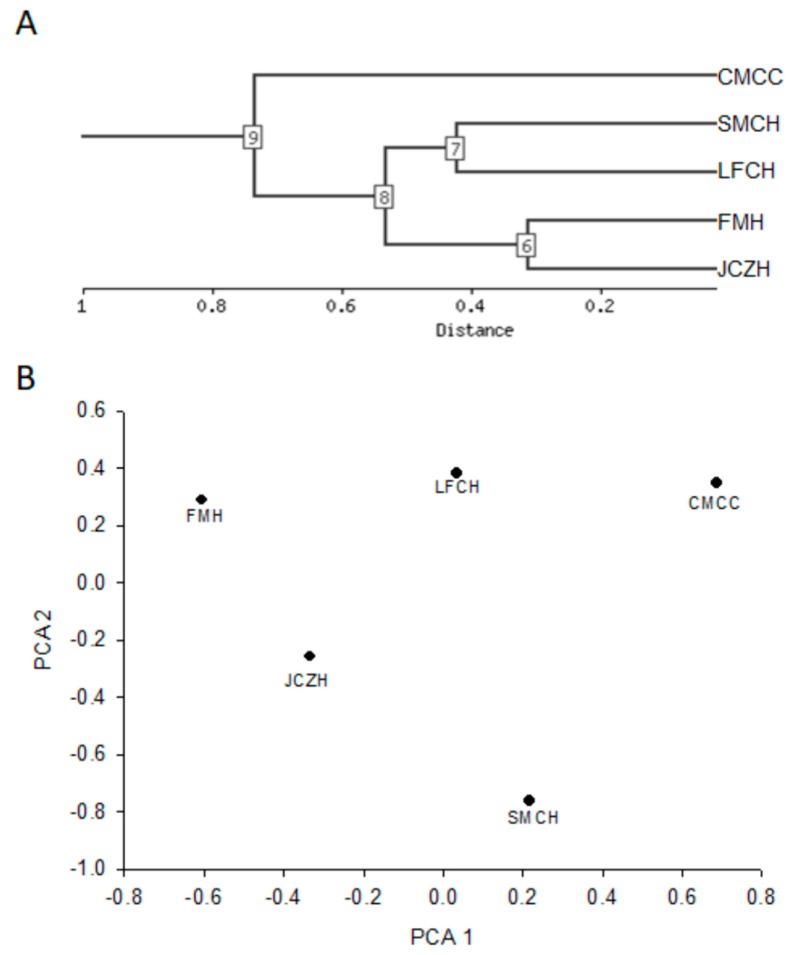
Associations between morphology and chemical composition of pine nut from five population of *P. cembroides* by hierarchical cluster analysis (**A**) and PCA (**B**), using the National Institute on Aging (NIA) array analysis tool. Population names are indicated in Table 4.

Morphometry and chemical composition data determined in this study were subjected to clustering analysis and principal component analysis in order to establish groups of populations and distances among them. NIA array analysis tool was used for hierarchical clustering and PCA analyses (Figure 3).

Both analyses showed that JCZH and FMH populations of the state of Hidalgo showed a substantial similarity between seed morphometry and chemical composition determined in this study, while SMCH and LFCH populations of the state of Hidalgo were clustered inside this group (Figure 3A). However, the CMCC population of the state of Chihuahua was the most distant. Principal components analysis also showed that the first two components explained 91.72% of the total variability, with 76.1% and 15.7%, respectively (Figure 3B). The variables that strongly contribute to PC1 were the antioxidant activity determined by FRAP assay and flavonoid and water content (Table 3). These results confirm that the phytochemical of pine nut grown in both states could be a significant influence on the separation of the population than morphological characteristics. JCZH and FMH population showed a tendency to present values higher in water content, total flavonoids, and antioxidant activity (Table 2 and Table 3).

## 3. Discussion

In this study, a statistically significant variation in the unshelled and shelled seed of different *P. cembroides* provenances has been observed regarding morphology (Table 1). Population variability with respect to pine nut morphometry analysis has been reported earlier in some *P. cembroides* communities. A study of the length, diameter, and weight of seed collected from Cadereyta in the state of Querétaro, Mexico, showed values similar to those found in our study [19]. Also, seeds production in the cones of *P. cembroides* presented variations in the number of seeds per cone, percentage of developed and aborted seeds, and percentage of germination associated with the year of harvest from Santa María Las Cuevas, Altzayanca, Tlaxcala, Mexico [22]. Our results showed that populations from Hidalgo State had the most prominent pine nut values associated with size and weight, while the population of Chihuahua State had the smallest ones. Some studies have previously indicated that bigger seed could be a desirable characteristic and a good indicator of success of planted forest trees in reforestation programs. Size correlates with increased higher root, shoot ratios, and seedling growth, which could improve the seedling performance by developing and maintaining a deep and extensive root system that can capture water from deep within the soil profile [23,24]. In this context, SMCH and JCZH populations of Hidalgo State can be considered the best source of seeds for a reproduction program in these zones.

The analysis of the chemical composition of pine nut from the five populations of *P. cembroides* showed an average of the water content of 14.6%. The moisture content of seed of *P. halepensis* (7.86%), *P. pinea* (8.47%), *P. pinaster* (8.23%), and *P. canariensis* (8.65%) have shown to oscillate slightly lower than those found in our study [9]. Loewe-Muñoz et al. [12] found an average of 5.6% of water content in seed collected of three different macrozones of *P. pinea* in Chile, which is lower than values determined in our study. Variation in water content of pine nut of *P. pinea* has been related to the weather condition of seed collection during the spring, immediately before cone maturation [25].

Ash content showed an average of 3.1%. These results were similar to those of the pine nuts of *P. pinea* (1.9%), *P. edulis* (2.7%), *P. koraiensis* (2.2%), *P. monophylla* (2.4), and *P. quadrifolia* (2.4%), but these were lower than those determined for pine nuts of *P. halepensis* (7.4%), *P. pinaster* (4.6%), *P. canariensis* (4.8%), and *P. maximartinezii* (4.3%) [9,10]. Loewe-Muñoz et al. [12] found an average of 4.4% of ashes in seed collected of three different macrozones of *P. pinea* in Chile, which is highest than values determined in our study.

Pine nut of *P. cembroides* could be a good source of essential fatty acid and hence a good source of dietary energy. Wolff and Marpeau [21] determined that the dehulled seeds of *P. cembroides* in the United States are a good source of oil (64%) with desirable fatty acid composition, including high levels of oleic (47%) and linoleic (41%) acids, but only 10% of saturated acids. Sagrero-Nieves [20] found that seed coat of pine nut of several phenotypes of *P. cembroides* from the central region of Veracruz showed differences in myristic (3.4–9.1%), oleic (36.7–47.2%), and linoleic (32.9–44.5%) acids. Total lipids determined in this study were higher compared to those determined for *P. pinea* (36.7%), *P. halepensis* (19.7%), *P. pinaster* (24.1%), and *P. canariensis* (23.9%) located from North of Algeria [9]. Furthermore, total lipids were higher compared with the determined for the species of *P. monophylla* (23%), *P. maximartinezii* (42.5%), and *P. quadrifolia* (37%) species [10]. Loewe-Muñoz et al. [12] found an average of 40.2% of lipids in seed collected of three different macrozones of *P. pinea* in Chile, which is lower than values determined in this study, and these variations can be due to the soil composition between regions and with the species [8,11].

In comparison with other species of pine nuts, the protein content was similar to that previously reported for the species of *P. pinaster* (16.2%), *P. koraiensis* (17.0%), *P. sibirica* (17.0%), and *P. canariensis* (16.7%). However, although the value remains within what is mentioned in the literature, these values were lower than those determined for the species of *P. halepensis* (26.6%), *P. maximartinezii* (31.3%), and *P. sabiniana* (28%) [9,10], but protein content of the pine nut of *P. cembroides* of the state of Hidalgo and Chihuahua was higher compared for the species of *P. pinea* (14.25%), *P. edulis* (14.3%), *P. monophylla* (9.5%), and *P. quadrifolia* (11%) [10]. Loewe-Muñoz et al. [12] found an average of 35.5% of protein content in seed collected of three different macrozones of *P. pinea* in Chile, which is highest than values determined in this study. These results have shown that the amount of proteins of pine nut, varies significantly; these variations could be the results of differences in soil composition between regions and depending on the species of pine [8,11].

Carbohydrate content determined in this study were higher than those reported for the species of *P. edulis* (18.1%), *P. koraiensis* (12%), *P. maximartinezii* (2.4%), *P. pinea* (6.5%), *P. sabiniana* (8%), and *P. sibirica* (12%), but were lower than those determined for the seeds of *P. monophylla* (54%) and *P. quadrifolia* (45%) [10]. These results suggest that the amount of carbohydrates content of pine nut varies significantly; these variations could be the results of differences in soil composition between regions and depending on the species of pine [8,11,26].

Phenolic and flavonoid compounds are an essential group of bioactive molecules found in plant seeds and exhibit various biological activities when these are included in the diet. Phenolic content of pine nut of *P. cembroides* showed an average of 5.5%. Although there are no previous results for this species, the results determined in this study were lower than those ones detected in pine nut of *P. pinea* (7.99 mg/g), *P. pinaster* (9.23 mg/g), *P. canariensis* (9.67 mg/g), *P. sibirica* (266 mg/g), and *P. koraiensis* (264 mg/g) [9,11,18,27], but were higher than those determined from seeds of *P. halepensis* (3.71 mg/g) [9]. Flavonoid content showed an average of 3.1%, these results were similar to those reported for *P. pinea* (2.17 mg/g), while these were higher than those determined in pine nut of *P. canariensis* (0.75 mg/g), *P. halepensis* (0.80 mg/g), and *P. pinaster* (1.42 mg/g) [9].

In this study, the total antioxidant activity was carried out using the 2,2-diphenyl-1-picrylhydrazyl (DPPH), ferric reducing antioxidant power (FRAP) and 2,2-azinobis-(3-ethylbenzothiazoline-6-sulfonic acid) (ABTS) radical scavenging assays. These methods are based on the same mechanism of propensity to donate hydrogens [28]. Our results showed a variation in the averages according to the population and the method used to determine these chemical compounds. The results showed that FMH population of the state of Hidalgo presented the highest activity, while the population of SMCH presented the lowest antioxidant capacity determined by DPPH and ABTS methodologies, and CMCC by FRAP assay. Some studies have established that the presence of phenolic and flavonoid contents in an extract could be related to a high antioxidant capacity. Our results showed a negative correlation between the total phenolic content and DPPH (r = −0.81, *p* = 0.09), ABTS^++^ (r = −0.452, *p* = 0.44), and FRAP (r = −0.442, *p* = 0.45). These results were opposite to those ones found in pine nut of *P. gerardiana*, where total phenolic content exhibited significant positive correlation with DPPH (r = 0.867), ABTS^++^ (r = 0.854), and FRAP (r = 0.934) [29]. However, when flavonoid content was correlated with the antioxidant activity ABTS (r = 0.87, *p* = 0.05), it was highest than FRAP (r = 0.83, *p* = 0.08) and DPPH (r = 0.76, *p* = 0.13). In addition, when the methods used to determine the antioxidant activity were correlated with each other, FRAP and ABTS (r = 0.99, *p* = 0.001) were higher than FRAP and DPPH (r = 0.563, *p* = 0.32), and DPPH and ABTS (r = 0.533, *p* = 0.35). Negative correlations indicate that the antioxidant capacity of most samples does not depend only on the content of phenolic compounds. Previous phytochemical analysis of the pine nuts composition has shown the presence of phytosterols, carotenoids, tocopherols, vitamin C, and polyunsaturated fatty acids, mainly linoleic acid; all of them with proven antioxidant capacity, whose concentrations exceed that of phenolic compounds [13,29,30]. Thus, given the high relation between the antioxidant activity determined by ABTS assay and the flavonoid content, these compounds content could be a good indicator of the presence of antioxidants compounds for pine nut of *P. cembroides*. In addition, the measurement of the antioxidant activity could be determined by using FRAP and ABTS assays in *P. cembroides*.

Some studies have shown that seed morphology and chemical composition variation could reflect the environmental conditions of the location where the *Pinus* species grow. Our result showed that latitude, longitude, and mean monthly maximum and minimum temperature were the most important climatic variable for unshelled and shelled pine nut characteristics (Figure 1A–E). These results agreed with Loewe-Muñoz et al. [12], who found that temperature (i.e., average summer temperature, maximum summer temperature, maximum average temperature, and thermal oscillation) was the most important climatic variables for cone, in-shell pine nut characteristics, and kernel weight variation across Chilean macrozones. Furthermore, our result showed that monthly maximum temperature showed an inverse correlation with total lipids (Figure 2A) and these were positively correlated with carbohydrate contents (Figure 2B). Lutz et al. [13] showed that ash, protein, and lipid contents exhibited significant correlation with the temperature of seed origin of *P. pinea* from Chile forest areas. Some studies have demonstrated that phytochemical contents vary widely among and within the different nut genotypes. However, other variables as the harvesting and environmental stresses (i.e., starvation, infection, predation, and UV light) could modulate the capacity for phytochemical synthesis [30]. Our results showed that mean monthly temperature and mean monthly minimum temperature were positively correlated with flavonoids and antioxidant activity determined by DPPH assay. Our result partially agreed with those reported by Lutz et al., [13], who showed that α-tocopherol contents exhibited significant correlation with the temperature of seed origin of *P. pinea* from Chile forest areas. Therefore, these results support that variation in the chemical and phytochemical composition of pine nut could be related to factors associated with (i) climatic conditions (i.e., temperature and temperature oscillations), (ii) physiological condition of plant (i.e., ripening and desiccation), and (iii) sanitary condition (i.e., attacks from pests) [12,13,30]. The analysis of the protein profile of seed of *P. pinea* collected of 30 populations distributed along a climatic gradient in Chile forest areas also demonstrated that the differentiation among them was more dependent on the environmental factors than on the genetics [31]. The principal component analysis has shown that accumulated rainfall, hydric deficit, and minimum average temperature were the climate variables mostly related to the principal component, showing clear correlations between proteins and the geoclimatic environment [31]. Principal components analysis also showed that the populations varied significantly in water content, total flavonoid and antioxidant activity (Table 2 and Table 3). Lutz et al. [13] established that the content of fiber, ashes, moisture, protein, and lipids of pine nuts are useful to segregate the three Chilean macrozones of *P. pinea*.

## 4. Materials and Methods

### 4.1. Biological Material and Seed Collection

**Figure 4:**
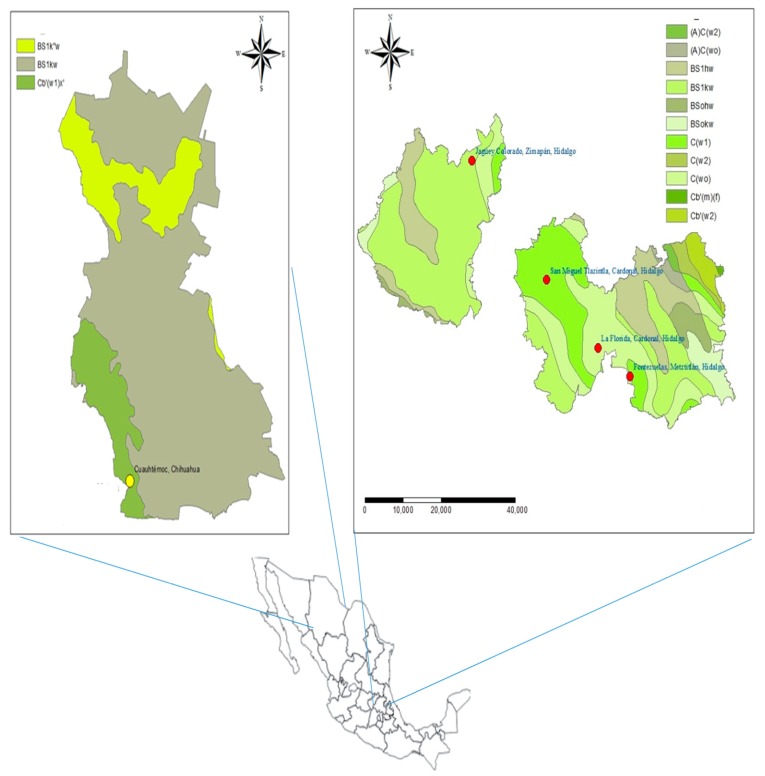
Distribution map of the five populations of *P. cembroides*. Source: Own elaboration from the set of climatic vector data from INEGI, 2013 (unit scale 1:100,000).

*P. cembroides* seeds were sampled in four populations located in the state of Hidalgo, Mexico and one located in the state of Chihuahua, Mexico (Figure 4 and Table 4).

In these localities, the populations of *P. cembroides* were associated with populations of *Quercus pringlei*, *Juniperus flaccida*, *Yucca filifera*, *Agave lechuguilla*, *Dasylirium longissimun*, *Chrysactinia mexicana*, *Flourensia resinosa*, *Eupatorium spinosarum*, *Mimosa biuncidera*, and *Opuntia rastreta*. The selection of the provenances was mainly done because of these localities are lands in which there is a traditional collection of pine nuts and these areas are in the process of evaluation to become germplasm producing zones. Table 4 shows the geographical and climatic characteristics of the provenances where the collection of the biological material was carried out. Climatic and geographical data were obtained from the database of the National Meteorological System of the National Water Commission from the historical period data of 1950 to 2010.

In each place, in order to collect the greatest variability of each of the sampled areas, 20 trees located 20 m from each other were randomly selected. The selection of the trees consisted of detecting healthy and vigorous trees. From each tree, ten cones distributed throughout the tree crown were collected. Cones were placed into an airtight polyethylene bag and immediately transported to the laboratory, where samples from each population were stored at room temperature until the cones were opened (20 days). Seeds were separated from the cones and stored in an airtight polyethylene bag at 4 °C.

### 4.2. Morphometric Analyses

**Figure 5:**
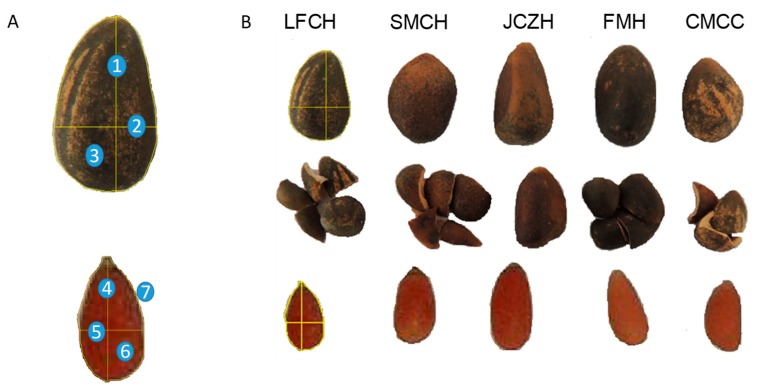
Morphology of the pine nut of *P. cembroides*. (**A**) Intact seed used to determine the weight of the seed. 1 = Vertical line used for the determination of the seed length. 2 = Horizontal line that represents the measurement used to determine the width of the seed. 3 = Measurement of the seed area. In the lower part, an intact scarified seed used to determine the weight of the megagametophyte. 4 = Vertical line used for megagametophyte length determination. 5 = Horizontal line that represents the measurement used to determine the width of megagametophyte. 6 = Area of megagametophyte. 7 = Perimeter of megagametophyte. (**B**) Morphology of the intact seed of La Florida, Cardonal (LFCH), San Miguel Tlazintla, Cardonal (SMCH), Jagüey Colorado, Zimapán (JCZH), Fontezuelas, Metztitlán (FMH), Malpaso Curves, Cuauhtémoc (CMCC). The intermediate part represents the seed coat and the lower part of the scarified seed.

To determine the variability in seed morphometric traits from each population, a total of 20 seeds were randomly selected and used for trait measurements (Figure 5).

Individual seed weight was determined using an analytical balance (Mettler Toledo AJ150, Ciudad de México, México). Length and width measurements were made on individual seed using a digital Vernier with precision of 0.01 mm (model CD: 15CP 500-181). To determine the weight of the seed coat and megagametophyte, 20 seeds were randomly selected and were scarified manually with the help of mechanical tweezers trying not to damage the megagametophyte (Figure 5A) and individual measurement of weight were measured separately using an analytical balance (Mettler Toledo AJ150, Ciudad de México, México) (Figure 5B). Then, megagametophytes were digitally photographed under a stereomicroscope, and the images were used to determine the area, perimeter, length, and diameter of the megagametophyte using an image analysis software (ImageJ, Bethesda, MD, USA) (Figure 5B).

### 4.3. Chemical Analyses

Prior to the proximate analyses, 50 seeds were randomly selected and pooled from each population, and were scarified manually using the methodology described previously. Once peeled, megagametophytes were weighed (AD^®^, model HR-250A) and triturated in a blade miller (Nutribullet^®^, mod. NB-101B, Los Angeles, CA, USA) until a homogeneous paste was obtained. The trituration of pine nut was performed at 4 °C for 5 min. In this condition, the proximate analyses were determined on three analytical replicates per each population and were determined according to the AOAC [31]. To determine the moisture percentage content 3 g of megagametophyte paste were weighted and then were dried at 105 °C in a ventilation oven for 8 h (VWR^®^, mod. 1324, Cornelius, OR, USA) (AOAC, 930.15). The ashes content was quantified by the incineration of the samples at 550 °C for 12 h to constant weight (Felisa^®^, mod. FE-340, Guadalajara, Jalisco, México) (AOAC, 923.03). The total protein content was performed by measurement of the total nitrogen using the Kjeldahl method (Labcono^^®^^, RapidStill II, Kansas City, MO, USA) (AOAC, 992.15). The total lipid content was carried out by the Soxhlet apparatus (Soxtec^®^, mod. 2043, Foss Analytical, Höganäs, Sweden) for 6 h using ethyl ether as solvent extractor (AOAC, 923.05). Total carbohydrates were determined by the difference method calculated as it is indicated in Equation (1) [31]:

Total carbohydrates = (100 − (total protein + total lipids + total minerals))(1)

### 4.4. Total Phenolics and Flavonoids

Seed standard extracts were obtained according to the methodology described by Kahkönen et al. [32]. Briefly, 0.3 g of lyophilized megagametophyte paste was weighed into a test tube and ten milliliters of 80% methanolic solution was added, stirred and sonicated for 30 min at 4 °C in the dark. Afterward, the extract was centrifuged (3000× *g*) for 10 min at 4 °C, and the supernatant was collected into a new test tube. Extraction was repeated and a total volume of 10 mL was finally completed. This extract was used to determine the total phenolics, flavonoids, and antioxidant activity.

Phenolic content was determined according to the methodology defined by Georgé et al. [33]. Briefly, 100 μL of standard extracts were taken into a test tube and 500 μL of the Folin–Ciocalteu reagent was added allowed to stand for 2 min. Thereafter, 400 μL of Na_2_CO_3_ were added. Then, the mixture was incubated at 50 °C for 15 min. Finally, the mixture was cooled in an ice bath and 250 μL were collected and placed in a microplate well. Absorbance was measured at 740 nm in a BioRad xMark Plus (Hercules, CA, USA), and data were obtained with the Microplate Manager 6.0 (Tokyo, Japan) computer software. A calibration curve was performed using gallic acid as a standard and results were expressed as mg gallic acid equivalents by 100 g of dry weight (DW) (mg GAE/100 g DW). All determinations were made in triplicate.

Total flavonoid content was determined according to the methodology defined by Georgé et al. [33]. Briefly, 31 μL of standard extracts were taken and placed in a well of a microplate. Then, 125 μL of distilled water and 9.3 μL of 5% NaNO_2_ (*w*/*v*) were added allowed to stand for 5 min. Thereafter, 9.3 μL of Al_2_Cl_3_ at 10% (*w*/*v*) was added. Then, the mixture was allowed to stand for 3 min and 125 μL of 0.5 M NaOH was added. Then, the mixture was incubated for 30 min in the absence of light and the absorbance was measured at 510 nm in a BioRad xMark Plus (Hercules, CA, USA), and the data were obtained with the Microplate Manager 6.0 (Tokyo, Japan) computer software. For quantification of the total flavonoid content, a calibration curve was performed using catechin as a standard and results were expressed as mg catechin equivalents by 100 g of dry weight (mg CE/100 g DW). All determinations were made in triplicate.

### 4.5. Antioxidant Activity

The total antioxidant activity was determined by 2,2-diphenyl-1-picrylhydrazyl (DPPH), ferric reducing antioxidant power (FRAP) and 2,2-azinobis-(3-ethylbenzothiazoline-6-sulfonic acid) (ABTS) radical scavenging assays using the methodology according to Thaipong et al. [34] and modified by Moreno-Escamilla et al. [35]. For DPPH assay, the solutions were prepared in 80% methanol. DPPH solution (60 mM) was prepared in methanol and was mixed in a 96-well microplate. Briefly, 25 μL of the standard extract was placed in a well of a microplate and 200 μL of the DPPH solution was added. Then, the mixture was incubated for 30 min at room temperature in absence of light and the absorbance was read at 517 nm every min for 1 h. TROLOX was used as standard and results were expressed as mmol TROLOX equivalents per 100 g of dry weight (TE/100 g DW). All determinations were made in triplicate.

The FRAP assay, 24 μL of the standard extract was placed in a well of a microplate and 180 μL FRAP reagent was added. Then, the mixture was incubated for 30 min at 37 °C and the absorbance was read at 595 nm every min for 30 min using a Bio-Rad microplate reader. TROLOX reactive was used as standard and results were expressed as mmol TROLOX equivalents per 100 g of dry weight (TE/100 g DW). All determinations were made in triplicate.

To determine the antioxidant activity by ABTS^++^ assay, 12 μL of the standard extract was placed in a well of a microplate and 285 μL of the ABTS reagent was added. Then, the mixture was incubated for 30 min at room temperature in absence of light and the absorbance was read at 734 nm using a Bio-Rad microplate reader. Results were expressed as the inhibition percentage of ABTS^++^. To determine the IC50 of ABTS^++^, TROLOX reactive was used as a standard and results were expressed as mmol TROLOX equivalents per 100 g of dry weight (TE/100 g DW). The IC50 was determined in the percentage inhibition plot with respect to the concentration of the sample, defined as the amount of the sample (mg/mL) necessary to obtain a 50% inhibition of the ABTS^++^ radical. All determinations were made in triplicate.

### 4.6. Statistical Analysis

Normality of frequency distributions was tested by the Kolmogorov–Smirnov test. A one-way ANOVA was performed to test for differences in morphometry and chemical composition of seed data. Pearson’s correlation was carried out to test for correlation between morphometry and chemical seed composition data. Statistical analysis was conducted using SPSS v.8.0 software (SPSS Inc., Chicago, IL, USA). The web-based software NIA array analysis tool [25] was used for cluster analysis of morphology and chemical composition by using and following the recommendations described by Sharov et al. [36].

## 5. Conclusions

This study presents the morphology and chemical composition of *P. cembroides*, which complement the chemical composition previously reported. The results confirm that seeds of SMCH and JCZH population of Hidalgo State could be considerate to be used as seed selection for a reproduction program, because these provenances had the longest, widest, and heaviest pine nuts. Seed chemical composition obtained in this study also demonstrate that JCZH and FMH population of Hidalgo state had the highest content of water, lipids, protein, flavonoids and antioxidant activity, while CMCC population of Chihuahua State presented the highest contents of ash and carbohydrates. Furthermore, temperature (i.e., mean monthly maximum and minimum temperature) could be the most important climatic variables for seed morphology and chemical composition. The seed of *P. cembroides* could be a rich source of important nutrients that appear to have a very positive on human health. In the future, a multiyear study should be done taking into account the measurement of several years of seed collection, as well as the correlation with the soil, climate, and geographic data, in combination with the analysis of lipid, amino acid, and protein profiles could help us to make a more robust data set and corroborate this hypothesis most strongly.

## Figures and Tables

**Table 1:** Seed morphology from five populations of *P. cembroides*.

Seed Morphology	SMCH	JCZH	FMH	LFCH	CMCC
Seed weight (g)	0.59 ± 0.03 ^a^	0.61 ± 0.02 ^a^	0.50 ± 0.02 ^b^	0.50 ± 0.02 ^b^	0.37 ± 0.01 ^c^
Seed length (mm)	15.4 ± 0.3 ^a^	15.2 ± 0.7 ^a, b^	14.2 ± 0.2 ^b, c^	14.0 ± 0.2 ^c^	12.5 ± 0.2 ^d^
Seed width (mm)	9.0 ± 0.2 ^a^	8.9 ± 0.1 ^a^	8.3 ± 0.2 ^b^	8.3 ± 0.2 ^b^	8.1 ± 0.2 ^b^
Seed coat weight (g)	0.43 ± 0.02 ^a^	0.42 ± 0.02 ^a^	0.32 ± 0.02 ^b^	0.30 ± 0.01 ^b^	0.25 ± 0.01 ^c^
Megagametophyte weight (g)	0.16 ± 0.01 ^a^	0.19 ± 0.01 ^a^	0.18 ± 0.01 ^a^	0.16 ± 0.02 ^a^	0.12 ± 0.01 ^b^
Megagametophyte length (mm)	12.4 ± 0.3 ^a^	12.1 ± 0.3 ^a, b^	11.6 ± 0.3 ^a, b^	11.35 ± 0.2 ^b^	10.5 ± 0.3 ^c^
Megagametophyte width (mm)	5.9 ± 0.1 ^a^	5.8 ± 0.1 ^a, b, c^	5.9 ± 0.2 ^a, b^	5.5 ± 0.2 ^b, c^	5.4 ± 0.1 ^c^
Megagametophyte area (mm^2^)	57.8 ± 1.8 ^a^	56.3 ± 2.0 ^a^	56.2 ± 1.9 ^a^	50.4 ± 2.3 ^b^	47.1 ± 1.7 ^b^
Megagametophyte perimeter (mm)	32.3 ± 0.6 ^a^	31.9 ± 0.8 ^a^	31.9 ± 0.8 ^a^	31.3 ± 1.4 ^a^	34.2 ± 3.2 ^a^

Descriptive statistics are presented in terms of the mean ± SD (n = 20). Mean values with the same letters indicate homogeneous subsets for *p* ≤ 0.05 according to Duncan test.

**Table 2:** Chemical composition, total phenolics, total flavonoids, and antioxidant activity from five population of *P. cembroides* seeds. The descriptive statistics are presented in terms of the mean ± SD (n = 3). Mean values with the same letters indicate homogeneous subsets for *p* ≤ 0.05 according to Duncan test.

Chemical Composition	FMH	JCZH	SMTH	LFCH	CMCC
Water content (%)	29.9 ± 0.1 ^a^	19.2 ± 0.1 ^b^	11.1 ± 0.2 ^c^	9.0 ± 0.1 ^d^	3.7 ± 0.1 ^e^
Ash content (%)	3.3 ± 0.0 ^b^	3.1 ± 0.0 ^c^	3.0 ± 0.0 ^d^	2.8 ± 0.0 ^e^	3.4 ± 0.0 ^a^
Total Lipids (%)	53.3 ± 0.6 ^a, b^	58.4 ± 0.2 ^a^	53.8 ± 3.2 ^a, b^	52.8 ± 3.1 ^a, b^	48.4 ± 3.0 ^b^
Protein content (%)	17.7 ± 0.1 ^a, b, c^	19.2 ± 0.1 ^a^	18.1 ± 0.4 ^a, b^	15.7 ± 0.1 ^c^	16.7 ± 1.3 ^b, c^
Carbohydrates (%) *	25.8 ± 0.6 ^a, b^	19.3 ± 0.2 ^a^	25.1 ± 3.5 ^a, b^	28.6 ± 3.1 ^a^	31.5 ± 4.1 ^a^
Total phenolic **	4.8 ± 0.1 ^c^	4.9 ± 0.1 ^c^	7.8 ± 0.1 ^a^	4 ± 0.1 ^d^	5.9 ± 0.2 ^b^
Total flavonoids ***	4.8 ± 0.1 ^a^	3.6 ± 0.1 ^b^	1.9 ± 0.0 ^c^	3.6 ± 0.1 ^b^	1.5 ± 0.0 ^d^
DPPH assay ****/% inhibition	66.6 ± 0.9 ^a^/71.1	64.3 ± 1.5 ^a, b^/78.2	63.4 ± 0.7 ^b^/74.3	66.4 ± 0.8 ^a^/78.4	64.4 ± 0.5 ^a, b^/75.8
FRAP assay ****	26.2 ± 0.1 ^a^	24.3 ± 0.5 ^b^	22.0 ± 0.3 ^c^	22.5 ± 0.1 ^c^	21.9 ± 0.2 ^c^
ABTS assay/% inhibition	28.2 ± 0.3 ^a^/14.3	18.9 ± 0.2 ^b^/23.9	10.9 ± 0.2 ^d^/5.3	12.6 ± 0.2 ^c^/7.2	12.3 ± 0.3 ^c^/6.8

Proximate analysis reported in dry basis (%, g/100 g of pine nut). * Calculated by difference; ** mg GAE per 100 g sample in dry weight; *** mg CE 100 per 100 g sample in dry weight, **** mmol TE per 100 g sample in dry weight.

**Table 3:** Seed characteristics that strongly contributed to total variability in the principal analysis components.

Characteristics	Log10 Change	Correlation	PC Number	Direction	LFCH	SMTCH	JCZH	FMH	CMCC
FRAP assay antioxidant activity	−0.36	−0.85	1	Negative	1.09	1.03	1.27	1.45	1.08
Flavonoids content	−0.52	−0.94	1	Negative	0.55	0.27	0.55	0.67	0.17
Water content	−0.87	−0.97	1	Negative	0.95	1.04	1.28	1.47	0.56

**Table 4:** Geographical and climatic data of the five populations of *P. cembroides* included in this study.

Population	Latitude/Longitude	Altitude (m)	Annual Precipitation (mm)	Mean Monthly Temperature (°C)	Mean Monthly Maximum Temperature (°C)	Mean Monthly Minimum Temperature (°C)	Climate Data
La Florida, Cardonal, Hidalgo (LFCH)	20°31′59.59″/98°58′48.8″	1953	555.6	15.3	22.6	8.0	El encino (00013151)
San Miguel Tlazintla, Cardonal, Hidalgo (SMCH)	20°39′20.0″/99°06′4.5″	2300	482.2	13.7	21.1	6.3	Santuario (00013070)
Jagüey Colorado, Zimapán, Hidalgo (JCZH)	20°52′10.5″/99°16′37.5″	2275	1051.0	13.8	20.6	7.1	Encarnación (00013065)
Fontezuelas, Metztitlán, Hidalgo (FMH)	20°28′56.5″/98°54′17.5″	2455	555.6	15.3	22.6	8.0	El encino (00013151)
Curvas de Malpaso Cuauhtémoc, Chihuahua (CMCC)	28°24′25.0″/107°0′06″	2242	447.6	14.7	23.7	5.6	Cuauhtémoc (00008026)
